# A Survey of Alzheimer’s Disease Early Diagnosis Methods for Cognitive Assessment

**DOI:** 10.3390/s20247292

**Published:** 2020-12-18

**Authors:** Juan Manuel Fernández Montenegro, Barbara Villarini, Anastassia Angelopoulou, Epaminondas Kapetanios, Jose Garcia-Rodriguez, Vasileios Argyriou

**Affiliations:** 1 Department of Networks and Digital Media, Kingston University, London KT1 2EE, UK; J.Montenegro@kingston.ac.uk (J.M.F.M.); Vasileios.Argyriou@kingston.ac.uk (V.A.); 2 Department of Computer Science, University of Westminster, London W1W 7BY, UK; B.Villarini@westminster.ac.uk (B.V.); A.Angelopoulou@westminster.ac.uk (A.A.); E.Kapetanios@westminster.ac.uk (E.K.); 3 Institute of Computing Research, University of Alicante, 03690 Alicante, Spain

**Keywords:** Alzheimer Screening, dementia, Virtual Environments, cognitive tests, behaviour analysis

## Abstract

Dementia is a syndrome that is characterised by the decline of different cognitive abilities. A high rate of deaths and high cost for detection, treatments, and patients care count amongst its consequences. Although there is no cure for dementia, a timely diagnosis helps in obtaining necessary support, appropriate medication, and maintenance, as far as possible, of engagement in intellectual, social, and physical activities. The early detection of Alzheimer Disease (AD) is considered to be of high importance for improving the quality of life of patients and their families. In particular, Virtual Reality (VR) is an expanding tool that can be used in order to assess cognitive abilities while navigating through a Virtual Environment (VE). The paper summarises common AD screening and diagnosis techniques focusing on the latest approaches that are based on Virtual Environments, behaviour analysis, and emotions recognition, aiming to provide more reliable and non-invasive diagnostics at home or in a clinical environment. Furthermore, different AD diagnosis evaluation methods and metrics are presented and discussed together with an overview of the different datasets.

## 1. Introduction

Dementia is a clinical disease that is characterised by cognitive and emotional impairments. Common symptoms are the decline of memory, reasoning, language, and perceptual interpretation, which affect daily functioning and the quality of life. Different types of dementia are defined according to specific combination of these symptoms, and Alzheimer’s Disease (AD) is the most common type (62% of the cases) mainly affecting elderly people. However, the reported numbers are also increasing among people under 65 years old in the UK and worldwide [1]. One of the first noticeable symptoms among AD’s patients is the loss of episodic memory and the difficulties of learning new information. As AD progresses, people experience greater memory loss, cognitive impairments, and behavioural changes. Typical problems include wandering and getting lost; being suspicious about family and caregivers; taking longer to complete daily tasks; or, not being able to speak, write, and walk properly [2].

Currently, when Alzheimer’s disease is diagnosed, the neuronal damage is spread enough to make it irreversible [3]. When neurons die, the other neurons do not divide and replace them, as other cells do, so the damage cannot be reversed [2]. Therefore, it is important to detect dementia at its very early stages in order to reduce the deterioration speed [4,5,6]. Early detection is also important, so the patient receives proper treatment and, therefore, increase their quality of life [7].

The cost of dementia is another issue to take into consideration. The worldwide cost of dementia is $818 billion, and it will become a trillion by 2018 [8]. The cost of dementia in the UK estimated in 2013 was 26.3 billion pounds; 4.3 billion were spent on healthcare costs, of which around 85 million were spent on diagnosis [1]. Therefore, it is essential to develop affordable diagnosis and support tools to help limit the increasing cost that is associated with dementia. One of the proposed initiatives is focused on the implementation of e-health (the use of Information Communication Technology (ICT)) solutions to reduce the cost and to make the health systems and solutions universally accessible [9,10,11]. Arief et al. [8] presented the strengths of e-health tools, such as the improvement in accessing health-care services by senior citizens, their cost-effectiveness, and their efficiency in managing health resources.

Alzheimer’s symptoms are studied in order to improve the results of existent approaches or create novel and more accurate diagnosis tools relying on new affordable and publicly available technologies. Alzheimer’s detection methods are classified into two different categories: invasive and non-invasive. Invasive methods require obtaining data from the interior of the patient’s body through procedures, such as lumbar puncture or blood extraction. These invasive methods try to define potential biomarkers that prove to be an accurate indicator of Alzheimer’s [12]. Most of them are not always safe and comfortable for the patient, and are sometimes unbearably painful. On the other hand, non-invasive tests are harmless and more convenient during the diagnosis process.

More recently, virtual environment (VE) and virtual reality (VR) based approaches are considered for AD diagnosis due to the advances in computer technologies and the availability of new devices. Immersive VE technology presents numerous advantages also as research tool in psychology increasing experimental realism and allowing to perform impossible manipulations of the reality [13]. Several studies have demonstrated that VEs can be used for neurophysiological assessment [14,15] and new technologies, such as emerging head-mounted displays, multi-sensorial interaction devices, and three-dimensional (3D) smart technologies can facilitate medical tests and therapies [16]. The aim is to provide more reliable and non-invasive diagnostics at home or in a nursing home environment.

This survey reviews state of the art publications that are related to non-invasive Alzheimer’s Disease (AD) screening tests. We will provide a review of different AD diagnosis techniques to evaluate participants’ cognition impairments focusing on Virtual Environment approaches, behaviour analysis and emotion recognition. This paper will offer a complete revision and description of the current and relevant works in the field, and we will illustrate an overview on the data-sets that are publicly available for AD studies. The number of works and proposals to use this technology is increasing and it shows the impact of VR on health-related tools. Some of the results from the approaches that are based on VR environments are discussed and compared with traditional methods to demonstrate the beneficial use of VEs for the diagnosis of AD, providing accurate detection and high acceptance by the users. Furthermore, this work demonstrates and presents the new technologies on VR that offer solutions on AD diagnosis. Additionally, it overviews the frameworks and practices utilised by medical institutions and practitioners and provides frameworks to allow for their integration in Virtual Environments.

The remainder of this study is organised, as follows: an in depth overview of methods for AD screening are analysed in Section 2. The approaches are organised in cognitive and non-cognitive screening. In Section 3, a review of methods based on virtual environment is presented and discussed. Section 4 analyses different AD diagnosis evaluation techniques and the databases that are utilised by the researcher to assess their results. Important findings and the results from the approaches described in the previous sections are discussed in Section 5. Finally, Section 6 provides some concluding remarks.

## 2. AD Non-Invasive Screening Methods Analysis

Apart from the invasive/non-invasive classification, Alzheimer’s detection methods can also be classified as non-cognitive and cognitive tests. Cognitive tests encompass the methods that assess the patients’ cognition; these procedures are non-invasive as well as easy to implement. On the other hand, non-cognitive tests include all other methods that were used to detect and diagnose dementia.

### 2.1. Overview of Non-Cognitive AD Screening Methods

Focusing on non-invasive non-cognitive methods, several approaches are followed in order to detect Alzheimer’s disease in its early stages.

#### 2.1.1. Neuroimaging Techniques

**Figure 1:**
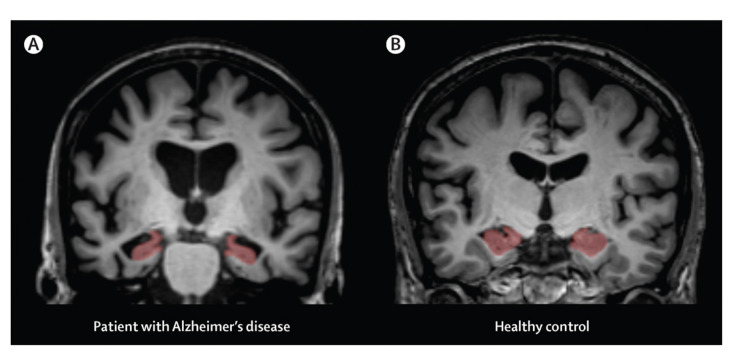
Magnetic Resonance Image (MRI) sample of a healthy person’s brain (**B**) and an Alzheimer’s patient’s one (**A**) [20].

Neuroimaging techniques, such as magnetic resonance imaging (MRI) or computed tomography (CT), are used to detect changes in the patients’ brains that are produced by the disease [2,17]. In the works presented in [17,18], the head MRI data of the patient is compared with the corresponding data of patients with Alzheimer’s by using machine learning techniques (see Figure 1). Agüera-Ortiz et al. [19] present a study using different MRI techniques, such as Diffusion Tensor Imaging (DTI) and Fluid-attenuated Inversion Recovery (FLAIR), in order to correlate the apathy of AD patients with changes in white and grey matter. As a result of their study, they found evidence of correlation between deficit of thinking (DT) and bilateral damage in the corpus callosum and internal capsule. Furthermore, they suggest that there is a relation between right-sided damage in the brain and apathy, which they associate with the purpose of the right hemisphere to initiate behaviour and monitor the environment. Nevertheless, since the acquisition of these images usually involves the use of medical equipment that is not easily accessible and the process is unpleasant (claustrophobic and noisy), these methods are not suitable for testing large groups of people due to cost and time. They are also not recommended for mental disease patients due to safety and discomfort reasons.

#### 2.1.2. Behaviour Analysis

Behaviour analysis approaches try to detect abnormal reactions to common circumstances or problems in daily living activities. The methods that assess cognition impairments in daily life activities usually require the use of external devices during a certain period of time in order to analyse patients’ behaviour [21,22]. For example, Aztiria et al. [22] placed a sensor on the patient’s foot in order to analyse their gait (step length and step height), since it reflects patients’ level of dementia. However, the gait measurement method still has some drawbacks; for instance, the patient has to wear a device for long periods of time. and the results obtained, despite being promising, are still not useful, since they have not been tested in Alzheimer patients.

Abe et al. present, in [4], another detection method that uses sensors in the patients’ homes in order to identify certain events. This non-cognitive approach is non-invasive and it requires only the consent of the patient to install the sensors. The main issue with these methods is that the obtained results are not precise enough (detection rates under 75%) to provide an accurate technique for dementia diagnosis at early stages. Amongst the problems of these approaches, there is the requirement of the patients’ consent to install the sensors and security problems that can lead to an invasion of personal information [23]. Moreover, these methods’ results are not precise enough (less than 75% detection rates) to provide an accurate technique for dementia diagnosis at early stages.

Ishii et al. [24] propose a sensor-based system in order to automatically determine suspicion of dementia. They use a series of sensors that are connected to a cloud where the data are analysed and compared with participants’ personal behaviour information in order to provide a diagnosis. In order to analyse behaviour, they check memory loss in situations, such as forgetting to turn a faucet off or to take a shower, sleep disorders, and wandering, with an average accuracy above 80%. They evaluated this system in different scenarios, but not in AD patients.

Chong et al. [25] also propose an automated system for AD diagnosis that is based on sensors. They analyse the participants’ behaviour by studying the daily routine activities that they carry out at home. They measure the number of hours with high activity, the number of hours spent on the bed, and the number of repeated activities. They conclude that the system for automated screening diagnosis is promising, but the quality of the sensors should be improved and some personalised considerations should be taken, since, sometimes, the patients’ mobility is reduced by other reasons than AD.

Varatharajan et al. [26] introduce a method for AD detection while using wearable devices. They analyse participants’ gait by using some special motion detection devices which subjects wear on their feet. These devices collect a huge amount of data that describes the participants’ gait such as number of steps, walking speed, stride length or cadence. They identified abrupt changes in foot movement patterns using the middle level cross identification function. Due to the difference in walking speed over time of the participants, the authors propose the use of dynamic time warping (DTW) to align the signal in time and to classify AD and healthy participants’ gait. They compare the results in terms of sensitivity and specificity with other classifiers, such as Support Vector Machine (SVM), k-Nearest Neighbors (kNN), and Inertial Navigation Algorithm (INA). Their outcome validates the use of DTW for gait classification to diagnose AD.

#### 2.1.3. Emotion Analysis

Other behaviour related approaches will include emotion analysis. As stated in [27], the decline of social cognition is one of the Alzheimer’s symptoms; therefore, many proposals are focused on patient’s capability to recognise emotions [28,29]. On the other hand, other approaches analyse patient’s reactions to specific stimuli. Several methods have been proposed for human emotion recognition due to the importance of emotion recognition in different disciplines, such as neuroscience or psychology [30]. Several proposals try to analyse those reactions/emotions while using different information, such as Electroencephalogram (EEG), eye tracking data, audio, or facial gestures. Some dementias, such as Lewy Body dementia, result in a lack of facial expression; therefore, facial expression analysis would be inconceivable. However, these approaches could prove particularly useful in AD where cognitive decline is associated with an increased facial expressiveness [31].

Eye tracking approaches are modelled on how doctors usually perform physical examinations. Eye movement and reflexes are monitored as part of the patient’s mental state evaluation [2], since visuospatial functions and visual processes go into decline due to Alzheimer [32]. Therefore, some of the visual methods for evaluating the mental state of a patient include the measure of the reaction time to certain stimuli, the assessment of attention, and the evaluation of patient’s visual memory [32,33,34].

In addition, neurological impairments, such as Dementia, are a cause of social exclusion and unequal treatment in the Deaf population. This is due to clinicians’ limited awareness of the linguistic features of impairments due to their limited knowledge regarding British Sign Language (BSL) and the absence of screening and diagnostic tools for this population [35,36]. Neurocognitive screening that is designed for signers promises significant progress in the detection of impairments. The only existing screening test for this population uses video stimulus material and requires video-recording of responses, and it relies on the availability of clinical specialists with fluency in BSL for its administration and interpretation as part of providing a firm diagnosis of dementia based on processing differences in the manual/facial actions of the signers [37].

### 2.2. Overview of Cognitive AD Screening Methods

Based on [38,39] cognition is defined as the use of the information that has been previously collected by a person to make behavioural decisions. Cognition encompasses several domains, but this survey focuses on those that are affected by Alzheimer’s disease. The American Psychiatric Association defined six neurocognitive domains that are impaired by AD.

- Complex attention: sustained, divided, and selective attention, i.e., the capacity of maintaining attention to a stimulus, focusing on two stimuli at the same time or keeping attention to one specific stimulus while others are interfering.
- Executive function: planning, decision-making, working memory, responding to feedback, overriding habits, and mental flexibility.
- Learning and memory: immediate, recent, and very long-term memory. Recent and very long-term memory include autobiographical memory.
- Language: understanding and expression.
- Perceptual-motor: the recognition of figures by shape or colour (including face recognition) or hand-eye coordination.
- Social cognition: ability to identify other people’s emotions.

#### 2.2.1. Methods Based on Problem-Solving Tasks

In order to detect a cognitive impairment, the most popular methods that challenge participants’ cognition are those that are based on problem-solving tasks and questions. Such methods are the most commonly used by doctors. Cordell et al. present, in [40], a comparative study of these methods, including the MMSE, Mini-Cog test, or Saint Louis University Mental Status (SLUMS) [41]. The Mini Mental State Examination (MMSE) is one of the most well-known and used tests [42,43]. The MMSE consists of twenty tasks that cover ten domains: orientation, registration, attention, calculation, recall, naming, repetition, comprehension, writing, and construction. Amongst these domains, orientation and attention are those that count the most. Mitchell [43] concludes that the MMSE is adequate for screening Alzheimer’s disease in advanced stages, but not Mild Cognitive Impairment (MCI). He also describes some of the limitations that are included on the MMSE and other cognitive tests, such as the ceiling effect, since the MMSE is not complex enough for high IQ patients [42,43]. The Mini-Cog test is better than the MMSE for early AD detection [44]. The test is composed of two tasks that take around three minutes. The first one is a three-word recall task, whereas the second requires the participant to draw a clock [45]. Each correct recalled word from the first task provides one point and the correct drawing of the clock is one point. Cognitive impairment is diagnosed if either the first task score is 0 or the score of the first task is 1 or 2 and the second task is 0. SLUMS is composed of eleven tasks and a maximum score of 30 [46]. It takes approximately seven minutes and it assesses orientation, memory, reasoning, and executive functioning, amongst others. Szcześniak et al. [46] demonstrate the validity of SLUMS examination for Alzheimer’s disease screening. Its results are correlated with other examinations, such as the MMSE. In addition, its specificity and sensitivity are superior to the MMSE for Mild Cognitive Impairment (MCI).

#### 2.2.2. Methods Based on Visual or Auditory Tasks

Other examples of cognitive tests that directly challenge cognition involve visual or auditory tasks. The memorization of interacting objects in the Visual Association Test (VAT) [47] requires the visualization of images. First, six images that contain two related objects or animals are shown to the participants. Afterwards, a new set of six images (where only one of the objects/animals of the previous set appears) is shown and the participants have to recall the missing object/animal. The Dichotic Listening test (DLT) [48] is used in order to detect the prevalence of the right ear when sounds are memorised by Alzheimer’s patients. With this aim, two single-digit numbers are played on each ear of the participant at the same time, e.g., number 1 on the right ear and number 8 on the left ear. This procedure is repeated six times while using different numbers and, at the end, the participants have to recall all of the numbers. Healthy participants usually remember digits from both ears equally whereas right ear dominance is detected in AD patients. This test also assesses memory impairments. The results that are provided by these tests show high levels of accuracy and specificity. Nevertheless, it should be noted that these tests do not provide variations to reduce the ceiling effect.

There are methods that are focused on visual impairments, since visuospatial functions and visual processes decline due to Alzheimer [32,49]. These methods evaluate the mental state of patients through the measurement of their reaction time in response to certain stimuli, the assessment of attention, or the evaluation of the patient’s visual memory [32,33]. Pereira et al. [32] analysed different methods that use eye movement to determine visual impairments in Alzheimer patients. These methods compared the eye movements of healthy people and Alzheimer patients in terms of fixation duration, refixations, or saccade orientation. As a result, most of the studies revealed an increment of saccades, defects in fixations, and slow pursuit movements. Nevertheless, as suggested by Pereira et al. in [32], these studies fail to consider attentional impairments as a multi-domain concept and their results still need to be corroborated by future studies.

**Figure 2:**
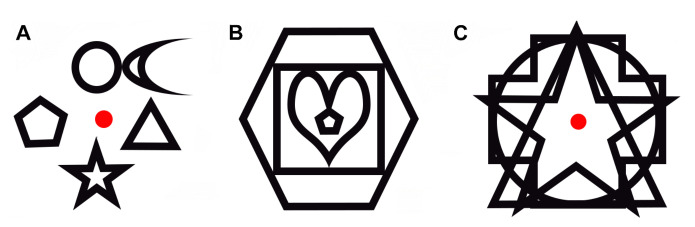
Example of the shapes presentation on each image during simultanagnosia assessment [50]: (**A**) adjacent, (**B**) embedded, and (**C**) overlapping.

Ruiz el al. [50] relate simultaneous object perception deficits to a visual processing speed impairment. In their research, they assess simultanagnosia, i.e., the inability to perceive more than one object at a time, and visual attention. In order to evaluate simultanagnosia, they use three tests, each one with their own specific tasks. The first test shows images of objects that are drawn with black lines on a white background. These objects are shown either individually or overlapping. During this test, both the identification speed and number of errors are analysed. The second test includes dot counting, position discrimination, and number location. The results depend on the number of correct answers, which are counted. As for the third test, it analyses the capacity to perceive different shapes drawn with black lines on a white background. The shapes are shown in four levels of difficulty: alone, adjacent, embedded, and overlapping (see Figure 2). During this test, the percentage of errors is analysed. Visual attention is assessed by asking the participant to recall as many letters as possible from an array of letters that is briefly presented. The outcome of these tasks allows for assessing visual processing speed and visual short-term memory storage. The overall outcome of these tests proved that the existence of simultaneous object perception deficits in early AD patients is linked to impaired visual processing speed.

Fraser et al. [51] classify MCI from healthy participants using gaze information extracted during two trials: reading of paragraphs silently and aloud. They use as features the information they obtain from saccades and fixations and they combine this data with information about the words which are used in the text, i.e., the duration of a fixation in a word whose number of repetitions in the text is low. First, they analyse the results of each trial independently, achieving better accuracy while using a Naïve Bayes classifier with the gaze features of the first trial. Finally, they combine both trials, achieving the best classification (86%) while using the Naïve Bayes classifier with the combination of words and gaze features. Despite their high accuracy, these results have not yet been validated in large groups. They also present the reading process differences between healthy and MCI patients. Healthy participants read the text from start to finish, whereas MCI participants tend to skip words and come back to them later. In addition, this difference is more evident when reading silently.

#### 2.2.3. Methods Based on Social and Emotional Tasks

Social cognition is one of the cognitive domains affected by Alzheimer. Associated symptoms include changes in behaviour or attitude, a decrease in empathy, or less ability to recognise facial expressions. Virtanen et al. [52] studied the correlation between the emotion recognition ability deterioration and the cognitive impairment level of older adults. Their participants group, aged 35 to 55 years, have to go through the MMSE and the Facial Emotion Recognition Test (FERT). The latest require the subjects to identify six basic emotions (anger, fear, disgust, sadness, happiness, and neutral). Each emotion, except neutral, is presented at three levels of intensity. A total of 160 images is shown to the participants. The emotion identification accuracy from FERT presented a strong correlation with MMSE. They also detected a curvilinear trend in all emotions but anger and happiness, which suggests that the detection of those emotions is more robust to cognitive deterioration.

Casal et al. [53] evaluates an AD screening tool. Affect-Gradior is a touchscreen application that requires the participants to identify seven basic emotions (including neutral) from ninety-one stimuli. During the test, the total score, the errors, and the processing time are measured. Their evaluation concludes that the Affect-Gradior scores and processing speed are correlated with other state of the art test (MMSE), being able to differentiate healthy from AD. In addition, they also probed that the results are not biased by age or participant mood.

Most research works focus on analysing whether AD patients are able to recognise facial expressions, but not many of them attempt to study the patients’ facial expressions in order to detect sudden mood changes or how they react to different stimuli. Fernández-Montenegro et al. [54] propose a method that is based on the patients’ reactions to autobiographical stimuli. Using Spontaneous Multimodal Database (SEMdb) they propose novel EEG features that are based on quaternion PCA to classify reactions to recent and distant autobiographical memories and reactions to known and known people. They only use four frontal sensors to reduce the EEG headset size. Their classification performance is measured while using the F1 score and it is compared with other EEG state-of-the-art methods. In addition, they also compared their results with facial expression recognition approaches that are based on landmark distances while using RGB images. Their overall results confirmed the superiority of FER techniques.

Fajtl et al. [55] present, in their work, a novel method for image memorability estimation that could be used to measure the memory decline of AD patients. Due to the importance of the visual attention component on the image memorability, the proposed AMNet architecture that contains a recurrent visual attention mechanism improves the overall performance providing human level accuracy.

## 3. Virtual Environment Based AD Screening Methods

Recently, Virtual Environments (VEs) have started to become part of medical tests and rehabilitation therapies due to the new advances in computer technologies [56]. Many approaches have been introduced to validate the use of virtual reality for neurocognitive assessment. In [57], the authors evaluate learning and memory cognitive abilities on healthy controls while navigating through a virtual environment using a head-mounted VR device. The first step of this experiment consists of showing ten objects that the participant has to memorise. Once memorised, they navigate through five areas of a Virtual Reality city, where they have to find the previously memorised objects. At the end of the experiment, the participants are asked to recall all of the objects and the areas where they have found them. The results are then compared with traditional neurocognitive methods, proving the correlation between them and validating the use of Virtual Environments for memory and learning assessment. The work that is presented in [58] assesses complex attention to healthy subjects (both military and civilian) and examines the ecological validity of virtual reality when using head-mounted VR devices in comparison to the less immersive experience of watching the scenario on a laptop screen wearing headphones. The participants are immersed in three low and three high intensity scenarios and they record psychophysiological information, such as startle eye blink amplitude and heart rate. In those scenarios, they also evaluate participants’ attention by showing a four-digit number in a central position during the first three scenarios and in a random position in the rest. Their results supported that a high level of immersion evokes a stronger physiological reaction than under low immersion conditions.

Cushman et al., in [59], also present a comparative study between a navigation system on VEs and the real world while evaluating Alzheimer’s participants’ navigation aptitudes and proving the ecological validity of VR environments. The navigational assessment test is divided into eight subtests. Firstly, a route demonstration is given in both real and virtual worlds; then, the subtests start. These subtests include route recall from ten landmarks, free recall of ten objects seen on the route, pointing to the location of ten objects from a certain point of the route, route-drawing on a map of the environment, route landmarks recall, photograph recognition, photograph location, and video location and direction. The results of the tests in both real and virtual scenarios were similar, therefore validating the use of Virtual Scenarios for navigation deficit assessment and screening of early AD.

In [60], Parson et al. analyse the appropriate way in order to create neurocognitive interfaces in VEs. In this work, they consider the possible loss of the experimental control that is related to VE fidelity. An increase of VE fidelity means a loss in control, so a balance should be found in relation with the test type. Psychophysiological computing is another interesting concept; this term refers to a technique that consists of monitoring people with a computer in order to create user-tailored applications. Currently, most of the psychophysiological evaluation is obtained after the VR experience, but new advances on the Brain Computer Interface (BCI) will allow for an automated and real time evaluation.

The work that was presented by Tarnanas et al. in [3,16] demonstrates that the use of VEs is beneficial when it comes to early dementia detection. It is possible to improve the results from previous cognitive tests, since floor and ceiling effects are reduced, which results in the creation of tests that can be adapted to the patients’ IQ. Furthermore, it is possible to increase immersion in the task that is in progress. Tarnanas et al. use large screens to display the environment as well as depth sensors in order to recognise the gestures of the patient’s body. This system requires the patient to move to the location where the equipment needed to carry out this test is available, due to the fact that its components are not portable or cost-effective, which makes it unsuitable for e-health applications.

**Figure 3:**
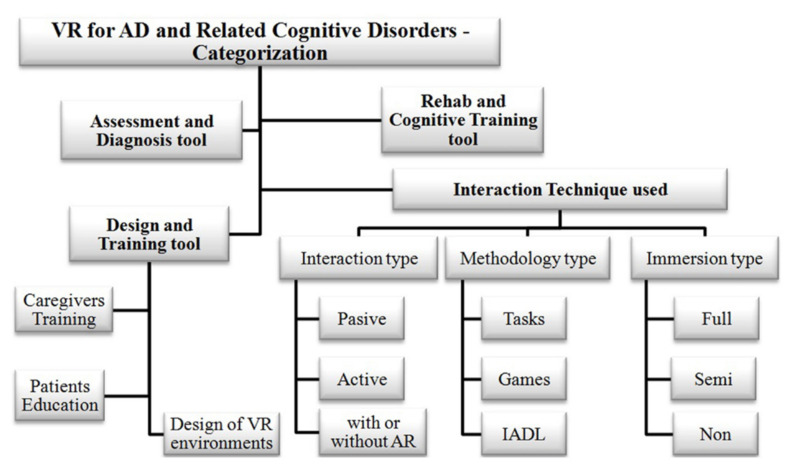
Garcia-Betances et al.’s [61] categorization of applications for Alzheimer Disease (AD) using Virtual Reality (VR).

The study conducted by García-Betances et al. in [61] shows the advantages of virtual environments for Alzheimer’s disease. In their work, they analyse the wide range of applications for AD that can be created while using VR. According to their analysis, as shown in Figure 3, the VR systems are classified according to some criteria. First, they consider the intended purpose, such as assessment and diagnosis, rehab and cognitive training, design, and training. Additionally, they classify VR systems that are based on the interaction technique used. Different methodologies are employed (e.g., tasks, games, Instrumental Activities of Daily Living (IADL)) and they are of different types, such as full-immersive, semi-immersive, non-immersive, and passive or active interaction. The authors point out the lack of immersion or interaction in most of the current virtual reality applications. They also mention that most of the approaches should be more affordable and accessible for home and nursing environments.

Vallejo et al. [62] evaluate an AD assessment tool that is based on serious games. This application evaluates several cognitive functions by using Virtual Environments on a PC monitor. Therefore, this is not considered to be full immersion. The researchers evaluate the performance of both healthy and AD participants, while these are doing everyday activities. The activities that they carry out are all related to shopping and cooking. First, they have to follow a series of arrows in order to get to the market and they then need to remember the way back. They will have previously memorised three ingredients that they need to buy in the market. Finally, when they are back home, they have to cook and set the table for two. The cognitive functions that are evaluated by this task include episodic memory, visuo-spatial orientation, recognition, spatial memory, executive function, and attention. As a result of their study, Vallejo et al. found considerable differences between the healthy and AD groups in the amount of time that they needed to fulfil the tasks as well as in their rate of success. Finally, the researchers conclude that serious games are a valid tool for cognitive impairment evaluation, even though it can be biased, as these activities are sometimes gender-oriented or culture-oriented.

Serino et al. [63] propose a training program while using Virtual Environments on a monitor for AD patients. Their program trains people to navigate in a virtual city, providing an interactive aerial view. The participants are in the middle of the city and they have to find objects and memorise their location (up to a maximum of three), one at a time (they cannot look for a new object until they have found the previous one). In the second stage, the participants are in the city again, but in a different place, and they have to find the locations of the objects that have been previously found in stage 1. To analyse cognitive capacity improvement, the participants are tested using the MMSE and neuropsychological battery that include test, such as verbal fluency or attentional matrices test. The researchers compared the evolution of patients that underwent a traditional recovery method and patients that had gone through their training program. The results revealed that the participants who had been trained on the VR program had improved more in terms of attentional abilities and spatial memory than those AD patients that were trained with traditional methods. Serino et al. consider that this is partly due to the use of the aerial views. Their results also suggest an improvement in executive functioning; however, due to the limited number of participants in the survey, further clinical trials are required to corroborate this.

Fernández-Montenegro et al. [64] proposed four novel tests on a virtual office, trying to increase the number of tasks that are concurrent in cognitive tests, in orderto reduce the ceiling effect and make them widely accessible. Their tests have been developed while using VR technology, such as VR glasses and mobile phones. The tests have been proposed when considering different cognitive domains: memory and learning and perceptual-motor and executive function and complex attention. Amongst the tasks, there is object memorization and recall, abnormalities detection, including one that requires the participant to perform simple movements (which are tracked with a Kinect sensor), sounds identification and association, and a test that is based on the inverse Turing problem. Instead of analysing how intelligent a computer is, the ability of the participant to discern real or incoherent information is evaluated. Their tests have been adapted to high IQ, increasing the number of subtasks and their difficulty according to the educational level of the participant.

Mohammadi et al. [65] introduce the Virtual Reality Navigation Task (VRNT) for amnestic Mild Cognitive Impairment (aMCI) and healthy participants’ differentiation. VRNT was tested on 110 participants, healthy and aMCI, where the latter was divided in single domain and multi domain aMCI by a neurologist using, amongst others, the Mini Mental State Examination score and the Boston Naming Test. The VRNT is designed with two virtual environments: a neighbourhood and a maze, to study allocentric and egocentric spatial navigation, respectively. The main difference between both environments is the lack of navigational landmarks in the maze. In order to fulfil the task, an overhead view of each environment is shown to the participants with the goal that they have to reach. Afterwards, they are moved to a first person view were they have to reach the goal. The number of correct responses and the response time was used to measure the subjects’ navigation performance. The results obtained using VRNT showed that single domain aMCI performance was worse than healthy for allocentric and egocentric navigation, with the difference being more clear in the second one. Nevertheless, it was not able to differentiate between single domain aMCI and healthy groups.

Foloppe et al. [66] present a single case study, where an old woman with probable AD was trained in four cooking tasks in real and virtual conditions in order to evaluate the potential of VR training. The participant was diagnosed probable AD according to the National Institute of Neurological and Communicative Disorders and Stroke and the Alzheimer’s Disease and Related Disorders Association (NINCDS-ADRDA) criteria and moderate dementia according to MMSE. Ten cooking related tasks were split into a sequence of actions that were transformed into oral and written instructions. All of the necessary elements for those actions were developed on a virtual environment that was shown in a monitor and controlled with a mouse. The participant had to choose four tasks amongst the ten, two of them were trained on VR, and the other two in real conditions. The process took one month, one week per task. The measures taken to evaluate the experiment included neuropsychological and functional assessment of each task before and after the training week. They also analysedwhetherif the changes that were produced by the training remained after six months. The results of this study demonstrated that training in a virtual environment can be as successful as a real environment training.

Eisapour et al. [67] created a VR exercising tool for people with MCI with the help of kinesiologists, recreational therapists, and Mild Cognitive Impairment (MCI) patients. The development process started with the analysis of the feasibility of creating a VR tool including VR glasses for MCI patients. Once the idea was conceivable, they started the design of the activities. They decided that a seated position will be the most appropriate and five motions were selected: head rotation, reaching straight ahead, cross-body reaching, lifting both arms, and rowing with both hands. The environment selected was a farm and the previous motions were linked with it, for example, grabbing fruits or picking flowers. After the first testing, they found out some problems due to the difference of mobility of the patients, so the tool needed a calibration task to adjust to each patient. As a result, the authors have created a promising tool for exercising MCI patients, despite some needed changes being recommended by the therapists.

## 4. Evaluation Techniques and Metrics for AD Diagnosis

In this section, different AD diagnosis evaluation techniques and metrics are analysed. Two main strategies were detected amongst the methods that evaluate participants’ impairments: methods that are based on machine learning classification and approaches based on score and threshold.

### 4.1. Machine Learning Techniques for AD Diagnosis

Machine learning is a part of artificial intelligence whose aim is to develop techniques that enable computers to learn. It aims to find relations between input variables and associated responses that allow for the prediction of responses to new input variables, as described by Rogers et al. [68]. Data from patients’ tests and tasks or medical data and their corresponding classes, such as healthy or non-healthy subject, could be utilised by machine learning techniques to find relations between data and labels. For example, ref [69] use SVM, Naïve Bayes and Optimum-Path Forest (OPF) classifiers to identify handwriting from healthy and Parkinson’s patients. For cognitive impairment screening purposes, many approaches use classification techniques that can be split into three groups, depending on the number of labels to be classified: one-class classification, binary classification, and multiclass classification. These techniques are mainly used by AD detection approaches that use large amounts of data for the classification. Amongst those approaches, we can find MRI and behaviour/sensor methods.

#### 4.1.1. Binary/MultiClass Classification

Binary and multiclass are the most frequently used classification types. The simplest classification problems are the binary ones, i.e., classifying data into two categories. Support Vector Machine (SVM) is one of the most popular binary classifiers, which was proposed for the first time in 1995 by Cortes et al. [70]. Multiclass problems require separating the given data into more than two categories. This problem can be solved either using multiple binary classifiers or a multiclass one. Akgul et al. [17] used MRI images as features of an SVM classifier in order to differentiate AD patients from healthy with an accuracy of 92%. In [26] the authors used DTW, SVM and kNN binary classifiers to categorise gait data from AD and healthy subjects. The work in [54] classifies recent and long distance memories while using EEG and facial images using SVM and Boosting classifiers. In [51], the authors use Naïve Bayes classifier to distinguish healthy from MCI patients while they are reading text paragraphs using features, such as gaze duration, saccade amplitude, and the total number of fixations. In [19], the general linear model is introduced to find correlations between MRI images and potentially AD explanatory variables. Vallejo et al. [62] use logistic regression binary classification per task while using the duration and correctness as features.

#### 4.1.2. One-Class Classification

One-Class Classification (OCC) aims to identify one type of objects and distinguish it from others when the data are very imbalanced (with training data mainly from one class). The data from this class are used as positive samples. There might also be data from other classes, but, due to factors, such as cost and ethics, it is difficult to obtain. These data will be treated as outliers. OCC methods can be organised into three families [71]. The first family is composed by density-estimation methods. These need a high amount of training data and they are not robust to outliers inside the training data. The second group deals with clustering-based methods. These consider the structure of the data and they are robust to outliers, but they require training data to represent the whole class. Finally, the third family includes methods that create a boundary around the desired class and the other ones. Their performance and robustness to outliers depend on the setting of this boundary. Rodionova et al. [72] mention other OCC methods for classification, depending on the use of outliers: compliant and rigorous. The latter gather all of the OCC classifiers that only use positive data for training, whereas the former add information that is given from some outliers to the model. When the data from the classes is overlapped it is generally more convenient to use compliant approaches.

One of the most appropriate applications of OCC is the automatic diagnosis of diseases [73], since, sometimes, it is difficult to obtain information from certain diseases due to the limited number of cases, to ethics, or cost. Lopez et al. [74] compare OCC and multiclass classifiers for the classification of audio signals for the early detection of Alzheimer. Speech-related Alzheimer’s symptoms include aphasia (speaking and understanding deficits) and Emotional Response problems. The authors have created a database, called AZTIAHO, which contains video recordings from fifty healthy and twenty AD participants. They use a Multi-Layer Perceptron (MLP) for the OCC and the multiclass classifier (MCC). OCC creates a model that represents the healthy group, whereas MCC models two classes, healthy and AD. The outcome of their experiments demonstrates that OCC outperforms the multiclass when AD data are scarce.

Das et al. [75] propose the use of OCC for the detection of errors in daily activities with the main purpose of helping people with dementia. They suggest the use of OCC classifiers with data extracted from sensors that are located in the patient’s home in order to provide help to both caregivers and patients when undertaking daily activities. Because it is impractical to define all of the possible errors that could occur during those activities, the researchers use the data from participants who completed the activities without making errors to train an OCC and the data with error is used for testing. Different features, such as number of sensors, event pause, or event time probability, are used to train the OCC. One Class Support Vector Machine (OCSVM) is the classifier used, which is a boundary method that finds a hyperplane that separates the positive class from the others. In addition, after the outliers from the healthy model are found, two other classifiers are proposed for error classification, one OCC and one MCC. The evaluation of the OCSVM shows a feeble performance, since the classifier detects many false positives. The authors attribute this to inaccurate error annotation. They also compare the use of OCSVM-OCSVM and OCSVM-MCC in order to detect errors and classify them; their classification results are similar.

### 4.2. Score/Threshold Based

Most popular AD screening methods, such as MMSE, SLUMS, and mini-Cog, are score based. These tests are formed by tasks that have an associated mark. By the end of the test, the participants are diagnosed as healthy or suspicious of AD if the added marks of each task (final score) is above a certain value (threshold). Following this approach, many other methods use the same evaluation system. For example, the methods that are proposed in [47,48] are methods that count the participants’ number of correct answers (the number of recalled image and numbers, respectively) and set a threshold to separate healthy from AD participants. The visual impairment related methods set thresholds in the number of fixations, their duration, time to identify stimulus, or number of errors/successful answers [32,49,50,51]. Approaches that analyse social cognition impairments (the capability of the patients to recognise emotions) count the number of correctly classified emotions and the time to detect an emotion [52,53]. Sensor/behaviour approaches use marks that are related to the number of actions forgotten, while the participants are doing daily activities. Chong et al. [25] used event related thresholds, which is, they settled a threshold that identifies an abnormal behaviour when an event happens more than a number of times or many events happen in a reduced amount of time. Abe et al. [4] use sensors that are located at patients’ home to detect specific events that could be forgotten by AD patients. The authors use a score based evaluation, where the events that are more probable to be forgotten receive a higher score than the others. Their approach diagnoses suspicion of dementia when the score is higher than a settled threshold after one week evaluation. When it comes to Virtual Reality approaches, they usually create experiences where the participant has to fulfil a number of tasks with an associated mark; therefore, the number of right answers, correctly completed tasks, and the time to fulfil them are usually measured. The authors in [64] created four different test divided in subtasks, each with an associated mark. Once one of the tests is finished, the total score and an evaluation chart is shown where the participants can check whether their score is inside the healthy range or if they need further evaluation. The proposed methods [57,63] scored the navigational and memory capability of the subjects. The participants had to memorise objects and locate them in a virtual scenario and recall them and their location at the end of the test. Parson et al. also used score evaluation in [58] to probe that different scores are obtained when performing the same tasks in different immersion conditions. Cushman et al. [59] used scores to mark each of their tasks and discriminant function analysis to select the tasks that most efficiently distinguished between healthy, MCI, and AD groups. Tarnanas et al. [16] created a score system combining four events that can happen during the set of activities that were included in the fire evacuation drill (omission of 1 activity, repetition of the same activity, incorrect performance of an activity, and the number of attempts to complete it) and the time spent to perform the tasks. Mohammadi et al. [65] also used the number of correct tasks and the execution time to categorise healthy and aMCI patients. In [66], the aim of Foloppe et al. was the evaluation of the training in VR and not the diagnosis of AD, but they also used a score based system to analyse the performance of the patient during a cooking activity and these scores where compared over time to measure patient’s improvement/learning rate.

### 4.3. Databases

**Figure 4:**
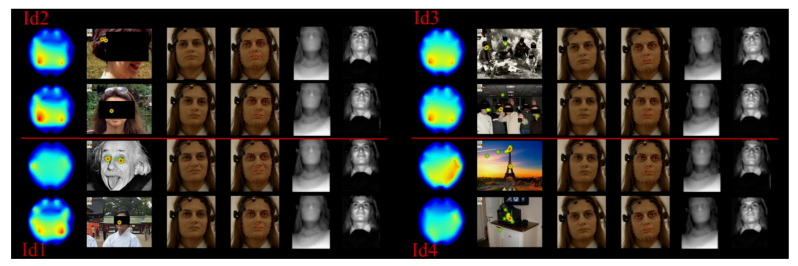
Different data modalities recorded from third task the SEMdb. The left figure shows, from top to bottom, (**Id1**) images of people from distant vs. recent past; and (**Id2**) famous vs. unknown people. The right figure shows from top to bottom (**Id3**) images of groups of people from distant vs. recent past; and (**Id4**) famous vs. unknown places [54].

In [76], Bell et al. identified 117 datasets that were related to dementia studies. Most of them contain clinical results from AD patients, such as blood test results, Magnetic Resonance Image (MRI) scans, or cognitive test results (the MMSE, primarily). For example, the Longitudinal Aging Study Amsterdam (LASA) [77] is a dataset, updated periodically, which contains information, such as emotional and cognitive interview-based surveys. It is based on a study of the ageing process experienced by the Dutch population and it contains information regarding cognitive impairments. The AlzGene database [78] contains genotype data pinpointing potential AD susceptible genes. The ADNI database is a longitudinal study that includes clinical data such as cognitive test results and biomarkers, MR images, positron emission tomography (PET) data, genetic data, and biospecimen data, such as blood, urine, or cerebrospinal fluid. BRAINnet [79] is one of the largest Alzheimer’s databases containing general and cognitive information from interviews and cognitive testing applications, EEG, ERPs, autonomic arousal measures, MRI, and genomics. Spontaneous Multimodal Database (SEMdb) [54] is a multimodal dataset for spontaneous emotional reaction recognition that contains multimodal information (HD RGB, depth and IR frames of the face, EEG signal, and eye gaze data) of nine healthy participants: five females and four males between 27 to 60 years old with different educational backgrounds that were taken while completing cognitive/visual tests. The general purpose of this dataset is AD screening. It contains recordings of participants while completing three tasks: gaze calibration, numeral search, and autobiographical tasks. The calibration info can be utilised for analysing patients’ complexity attention. The search task is based on an AD screening test that analyses search performance. The study of the number and duration of fixations allows for differentiating between AD and healthy participants. The main novelty of SEM database is enclosed in the third task (autobiographical). It provides spontaneous non-posed reactions to autobiographical and non-autobiographical visual stimuli data (see Figure 4).

## 5. Discussion

In this survey, AD screening methods have been reviewed. Non-cognitive approaches are usually uncomfortable, expensive and time-consuming. Therefore, they are not the most convenient for AD patients. Although there is merit in using these methods, cognitive tests remain the most commonly used in diagnosing AD. Table 1 provides a summary of the non-invasive AD screening methods that have been reviewed. It shows the type of approach (if cognitive or non-cognitive), the specific type of analysis, the devices that have been used, and a classification according to the invasiveness of the screening, the accuracy related to the assessment of cognitive impairments, and the cost and time effectiveness.

The MRI approaches are highly reliable tests for AD diagnosis; however, other approaches are necessary due to MRI cost and invasiveness, as shown Table 1. The other methods can be grouped in five main categories ordered by AD assessment effectiveness: VR, emotion, problem solving, visual and behaviour based tests. Some of the VR tests are the only ones that take into account the IQ of the patients in order to reduce the effects of the cognitive reserve making them the most promising for AD assessment [16,64]. The work in [16] has proved to be reliable, however the settings are not easily accessible, not cost efficient, and the tasks evaluated mainly executive functioning domain impairments. The approach that is followed by [64] is cost efficient and it evaluates different cognitive domains, but it has been tested with small amount of data. Amongst the emotion based methods, the capacity of patients to differentiate emotions is the approach that obtains better results [52,53]. The problem solving methods are still the most used approaches by doctors, since they are the easiest to use, despite that they do not take into account cognitive reserve. The most popular is MMSE, despite that some papers have probed other similar approaches, such as SLUMS or mini-Cog, provide better assessment. Visual related methods are easy to use and very helpful for analysing attention impairment, but the results that are obtained by those methods are not good enough to assess the disease. Finally, behaviour approaches did not provide accurate results and their implementation is usually difficult due to privacy invasiveness. Other types of methods exist, but they need to be further studied by researchers.

Furthermore, the table shows that most of the cognitive approaches have high accuracy when it comes to Alzheimer’s detection. Although, one of their weaknesses is the evaluation of brain’s capacity to compensate brain damage (cognitive reserve) [3,5]. Another well-known problem is the adaptability of the tests according to the patient’s IQ, since most of the tasks that integrate an Alzheimer’s detection cognitive test usually are too simple to evaluate intelligent patients. Therefore, it is necessary to create tests where the results are not correlated with the IQ of the patient. The use of computerised tests helps to create intelligence adaptable cognitive tests [3,80]. Moreover, based on the available technology, such as virtual environments (VEs), it is possible to design new types of tests and approaches. A relevant aspect regards the validation of tests that are based on VEs to demonstrate that they provide a valid alternative to traditional methods for assessing cognitive skills. The following section provides an overview of the techniques that are used to validate AD screening based on VR. The results and main findings show how VEs provide a new class of tools in support of the diagnostic assessment and cognitive training in AD.

### Validation Techniques and Results

In order to validate their results, most of the proposed methods firstly test their participants while using already validated state-of-the-art methods. MMSE, Activities of Daily Living (ADL) questionnaire, and MRI scans are some of the most popular comparative methods. The results obtained by the aforementioned methods are correlated using measures, such as Pearson’s and Spearman’s correlation coefficients. A correlation coefficient measures the extent to which two variables tend to change together. Pearson’s correlation measures the linear dependence of two continuous variables and the Spearman correlation evaluates the monotonic relationship between two variables [81]. These coefficients show a strong correlation between variables when the values are close to 1 or −1, but no correlation when they are close to 0. De Winter et al. [82] probed the suitability of Pearson’s correlation for light-tailed distributions and the Spearman’s correlation for heavy-tailed distributions or the ones that contain outliers, such as those present in psychological research.

Amongst the papers from the previous section, ref [64] utilised Pearson’s correlation to correlate their test results with state-of-the-art test, such as DLT, VAT, and SLUMS. Their results show high correlations demonstrating the effectiveness of using VEs to assess cognitive impairments. Tarnanas et al. [16] used the Spearman’s correlation between patients’ fire evacuation scores and MMSE and the Instrumental Activities of Daily Living (ADL) questionnaire ones. A ROC analysis was conducted for Virtual Reality Day-Out Task (VR-DOT), MMSE, ADL, and Rey Auditory Verbal Learning Test from amnestic-type mild cognitive impairment (aMCI) and AD groups. Probing the VR environment is able to classify healthy, MCI, and mild AD patients better than traditional methods, as shown in Table 2.

**Figure 5:**
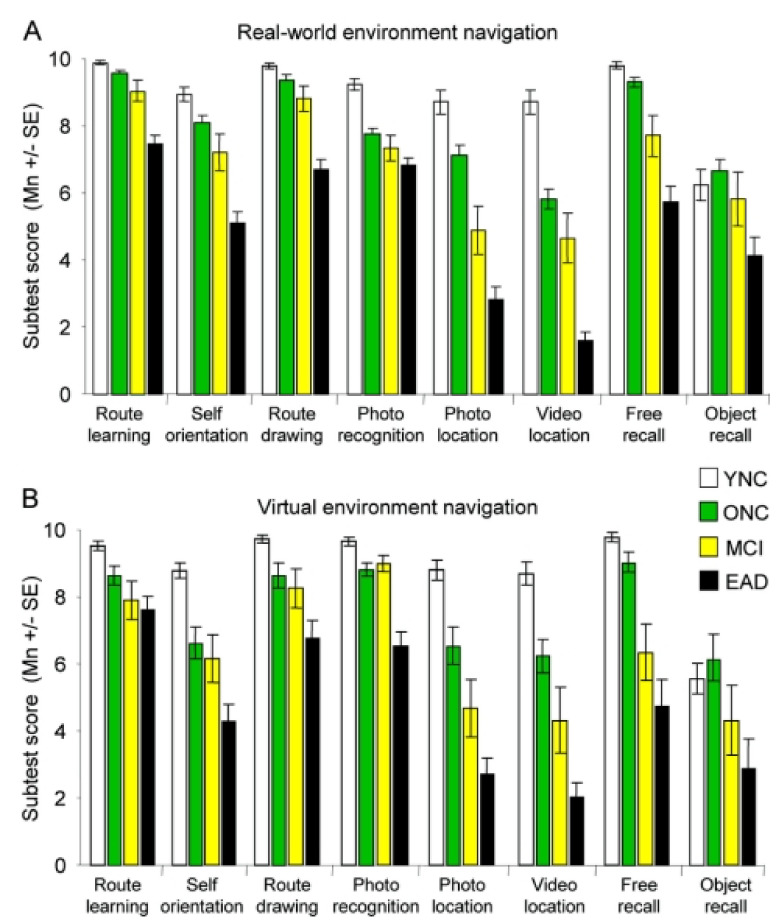
Across subject groups, the same pattern of relative performance was apparent on subtests, as was seen on total test scores; poorer performance from young normal controls (YNC) to older normal controls (ONC), to patients with mild cognitive impairment (MCI), and to patients with early Alzheimer disease (EAD). The real-world and virtual environments yielded similar patterns and magnitudes of differences across subject groups (young normal controls (YNC), older normal controls (ONC), patients with mild cognitive impairment (MCI), and patients with early Alzheimer disease (EAD)). (**A**) Real-world environment navigation; (**B**) virtual environment navigation. The photo and video location subtests yielded the lowest mean scores from both the MCI and AD groups [59].

Parsons et al. [57] used two-tailed Pearson’s correlation to compare their scores with traditional neuropsychological measures, such as the Hopkins Verbal Learning Test-Revised and the Wechsler Test of Adult Reading, in order to evaluate domains, such as verbal fluency, attention, and processing speed. Their findings suggest the consistency between VE approaches and the traditional one that is based on paper-and-pencil involving learning and memory. The authors in [59] prove the validity of VR to assess navigation skills evaluating AD participants capabilities to navigate real and VR environments. They utilised discriminant function analysis in order to identify the subtests that better distinguish healthy young, old, MCI, and early AD groups; obtaining similar results for tests on real and virtual environments (Figure 5). In addition, the authors used multivariate analyses of variance in order to evaluate the correlation of their proposed tests with the state-of-the-art neuropsychological ones.

Mohammadi et al. [65] utilised Pearson’s correlation coefficient in order to find the relationship between neuropsychological parameters and their results, with two-tailed analysis and significance level *p* < 0.05. Ruiz et al. [50] used Spearman analysis in order to demonstrate that their simultanagnosia tests’ results are correlated with MMSE ones, but not with the patients’ educational background or age.

The above validations results highlight the consistency and validity of the VR environments in assessing cognitive impairments. Furthermore, VEs provide additional advantages to traditional cognitive tests, since it is possible to immerse the patient in a controlled situation [16,61,64,83,84]. Parsons et al. [58,60,85] have demonstrated the ecological validity of VEs and their benefits, such as the precision of data retrieved by the computer and the better control of the environment.

Additionally, VR training could improve the condition and cognitive skills of AD patients. Vallejo et al. [62] checked which of their tasks better help to distinguish healthy and AD while using likelihood-ratio-test, showing that cooking and shopping failures are the best indicators of an AD related impairment.Additionally, they present the evolution of AD patient during training on four different cooking tasks. Two tasks were trained on a real scenario (prepare coffee and breakfast) and the other two on a VE (prepare cake and soup). Their results show the evolution of the participant on the different tasks. In particular, they show an increasing of 11.3% of autonomy from his baseline after training in a real scenario and and of 15.4% using virtual reality. Serino et al. [63] drew a similar conclusion. Their study showed the evolution of two groups of AD patients. One group used VR training and the other (control group) used standard training. The results in Table 3 demonstrate a similar evolution using both trainings in most of the evaluation categories, but the VR training outperforms the traditional in terms of attention skills.

## 6. Conclusions

This paper overviews state of the art methods for AD early detection focusing on cognitive and VE/VR based approaches that offer a complete panoramic of the current and relevant works in the field. Different AD diagnosis evaluation methods and metrics are presented and discussed, together with an overview of the different datasets. Furthermore, overviews of related VR technologies and frameworks are presented, offering solutions for practitioners integrating existing solutions in Virtual Environments and overcoming the ceiling effect and other limitations of the classic cognitive approaches for AD diagnosis. An overview of non-cognitive approaches is also presented, including biomarkers and MRI techniques. However, we focused on cognitive, due to the invasiveness and uncomfortability of many of non-cognitive approaches. On the cognitive approaches section, we gather all of the techniques that challenge patients’ cognition. Some of these approaches include behaviour, gaze, and EEG analysis, while the subjects perform cognitive tasks. The most commonly used methods are question-based and they only require pen and paper to be completed, due to their adaptability to IQ and the type of tasks limitations, computerized cognitive approaches are being considered. Virtual reality options are currently taken into consideration due to the diverse range of assessment tasks that can be created (including those ones that are complicated to recreate on a real scenario) and the high immersion of the participants during the diagnosis process. On the last section we discuss data analysis and evaluation approaches used in comparative studies. The trend for cognitive approaches is score based diagnosis. The validation of the scores that were obtained by novel methods is done by correlating them with already settled state of the art methods, such as MMSE scores. Finally, a summary of the different datasets used for AD studies is presented. Most of these databases contain MMSE and MRI data from healthy and AD patients. The impact of VR on health related tools is noticeable and the number of proposals to use this technology for treatment and diagnosis is increasing. Its use for cognition assessment allows for the evaluation of many AD affected cognitive domains while using only one tool. The state of the art methods have to be proved valid for AD diagnosis, providing accurate detections and a huge acceptance by the users.

## Figures and Tables

**Table 1:** Summary of AD screening methods. The classification is rated with one to three stars (*) and a mark (-) if it is not applicable.

Name and Reference	Type of Screening	Type of Method	Devices Used	Invasiveness	AD Assessment Precision	Cost and Time
Multimodal MRI study [19]	Non-cognitive	Neuroimaging technique	MRI scan, medical equipment	** (Claustrophobic and noisy process)	***	***
Gait analysis [22]	Non-cognitive	Behaviour analysis	Sensor on patient’s foot	** (Wearing the device for long period)	*	***
Behaviour sensing [4]	Non-cognitive	Behaviour analysis	Sensors in patient’s home	* (Invasion of personal information)	*	**
M2 M/IoT platform [24]	Non-cognitive	Behaviour analysis	Sensors in patient’s home connect to a cloud	* (Invasion of personal information)	- (Tested on elderly people)	**
IoT sensors study [25]	Non-cognitive	Behaviour analysis	Sensors in patient’s home	* (Invasion of personal information)	*	**
Dynamic time warping [26]	Non-cognitive	Behaviour analysis	Sensor on patient’s feet	** (Wearing the device for long period)	**	***
Eye tracking during avisual paired comparison [33]	Non-cognitive	Visual analysis	Eye tracking device	-	*	**
Visual search [34]	Non-cognitive	Behaviour analysis	Large computer screen	-	**	*
MMSE [42]	Cognitive	Problem-solving tasks	Simple objects	-	** (High precision for advanced AD, not for MCI)	*
Mini-cog test [44]	Cognitive	Problem-solving tasks	Pen and paper	-	***	*
SLUMS [46]	Cognitive	Problem-solving tasks	Simple objects	-	***	*
VAT test [47]	Cognitive	Visual tasks	Set of images	-	**	*
DLT test [48]	Cognitive	Listening tasks	Headphones	*	**	*
Object perception [50]	Cognitive	Visual tasks	Set of images	-	*	*
Eye movement during reading [51]	Cognitive	Visual and attention tasks	Eye tracking device	-	**	**
Recognition of emotions [52]	Cognitive	Emotional tasks	Set of images	-	***	*
Computer-based emotion recognition [53]	Cognitive	Emotional tasks	Touchscreen application	-	***	*
Autobiographical memories [54]	Cognitive	Emotional tasks	EEG device	**	-(To be tested on AD patients)	**
VEs for memory functioning [57]	Cognitive	VEs, problem solving, memory tasks	Head-mounted VR device	*	***	**
Navigational deficit detection [59]	Cognitive	VEs, problem solving, navigation tasks	Head-mounted VR device	*	***	**
Computerised test [16]	Cognitive	VEs, problem solving, navigation tasks	Large screens, depth sensors	-	***	**
Serious games tool [62]	Cognitive	VEs, problem solving, navigation tasks	PC monitor	-	* (Biased as the activities aregender and culture oriented)	*
Turing test and VEs [64]	Cognitive	VEs, problem solving, memory tasks	VR glasses, mobile phones	*	** (Tested on small dataset)	*
VRNT [65]	Cognitive	VEs, problem solving tasks	Monitors, VR devices	*	**	*
VR excercising tool [67]	Cognitive	Problem solving tasks	VR glasses	*	* (Needs some changesrecommended by therapists)	*

**Table 2:** Area under the curve (AUC) for standard neuropsychological test scores and Virtual Reality Day-Out Task (VR-DOT) for healthy controls versus patients with amnestic-type mild cognitive impairment (aMCI) and patients with aMCI versus patients with mild Alzheimer-type dementia (AD) [16].

Test	Healthy Control vs. aMCI	aMCI vs. Mild AD
	**AUC (95% CI)**	**AUC (95% CI)**
MMSE	0.79 (0.68,0.91)	0.99 (0.97,1.00)
Bristol scores ADL	0.75 (0.62,0.88)	0.88 (0.78,0.97)
Blessed score ADL	0.77 (0.64,0.89)	0.87 (0.79,0.98)
RAVLT delayed recall	0.82 (0.77,0.93)	0.88 (0.79,0.98)
DOT-VR	0.96 (0.88,0.99)	0.95 (0.88,1.00)

MMSE: Mini-Mental State Examination, ADL: Activities of Daily Living, RAVLT: Rey Auditory Verbal Learning Test.

**Table 3:** One group used VR training and the other (control group) used standard training.

	VR Group-AD	Control Group-AD
Verbal fluency test	−1.60(4.00)	0.20 (4.21)
Verbal categorical test	0.70 (4.71)	0.50 (4.21)
MMSE	0.84 (4.26)	−0.66 (1.94)
Attentional matrices test	4.71 (9.52)	0.70 (2.89)
Digit span test	0.01 (0.82)	−0.33 (0.90)
Corsi block test—span	0.17 (1.20)	0.14 (1.21)
Corsi block test—supraspan	1.56 (2.53)	−0.01 (1.43)

Delta scores obtained from the pre-post neuropsychological assessment for patients with AD assigned to the VR-based training (“VR Group-AD”) and for patients with AD assigned to the “Control Group-AD” (“Control Group-AD”). Data are shown as means and standard deviations (SD) [63].
